# Supplementary material for: VCP/p97-associated proteins are binders and debranching enzymes of K48–K63-branched ubiquitin chains
Source: Nat Struct Mol Biol. 2024 Jul 8;31(12):1872–87. doi: 10.1038/s41594-024-01354-y (PMC11638074; doi:10.1038/s41594-024-01354-y)
Supplement: Supplementary file 1 — Reporting Summary [file 41594_2024_1354_MOESM1_ESM.pdf]

Corresponding author(s): Yogesh Kulathu

Last updated by author(s): 2024/05/14

Reporting Summary

Nature Portfolio wishes to improve the reproducibility of the work that we publish. This form provides structure for consistency and transparency in reporting. For further information on Nature Portfolio policies, see our Editorial Policies and the Editorial Policy Checklist.

Statistics

For all statistical analyses, confirm that the following items are present in the figure legend, table legend, main text, or Methods section.

n/a	Confirmed	
☐	☒	The exact sample size (*n*) for each experimental group/condition, given as a discrete number and unit of measurement
☒	☐	A statement on whether measurements were taken from distinct samples or whether the same sample was measured repeatedly
☐	☒	The statistical test(s) used AND whether they are one- or two-sided<br>*Only common tests should be described solely by name; describe more complex techniques in the Methods section.*
☒	☐	A description of all covariates tested
☐	☒	A description of any assumptions or corrections, such as tests of normality and adjustment for multiple comparisons
☐	☒	A full description of the statistical parameters including central tendency (e.g. means) or other basic estimates (e.g. regression coefficient) AND variation (e.g. standard deviation) or associated estimates of uncertainty (e.g. confidence intervals)
☐	☒	For null hypothesis testing, the test statistic (e.g. *F*, *t*, *r*) with confidence intervals, effect sizes, degrees of freedom and *P* value noted<br>*Give P values as exact values whenever suitable.*
☒	☐	For Bayesian analysis, information on the choice of priors and Markov chain Monte Carlo settings
☐	☒	For hierarchical and complex designs, identification of the appropriate level for tests and full reporting of outcomes
☒	☐	Estimates of effect sizes (e.g. Cohen's *d*, Pearson's *r*), indicating how they were calculated

Our web collection on statistics for biologists contains articles on many of the points above.

Software and code

Policy information about availability of computer code

Data collection	Crystallography data were collected using the software listed in the methods. ITC data were collected using Malvern software: MicroCal ITC Analysis Software (v1.22.1293.0) and MicroCal ITC (v1.29.32). Microscopy data were collected using Zen Blue software (v2.6.76)
Data analysis	Western blot images were quantified using LiCor ImageStudio Lite v5.2.5 or BioRad ImageLab v6.1.0, and subsequently analysed using Microsoft Excel for Mac (Office 365, various versions) and Prism 9 (v9.5.1) for macOS.<br>Custom python scripts were used to analyse the DIA mass spec and ULTIMAT DUB assay data and have been deposited to Zenodo (10.5281/zenodo.11204921). Peptide searches were conducted using DiaNN (v1.8.0). Statistical analysis for DIA mass spec was carried out in Perseus (v1.16.15.0).<br>Microscopy images were stitched using an ImageJ macro (ImageJ v1.54b). Image analysis was conducted using CellTool (v1.6.0.4). R scripts used for image data processing have been deposited to Zenodo (10.5281/zenodo.11204921).

For manuscripts utilizing custom algorithms or software that are central to the research but not yet described in published literature, software must be made available to editors and reviewers. We strongly encourage code deposition in a community repository (e.g. GitHub). See the Nature Portfolio guidelines for submitting code & software for further information.

Data

Policy information about availability of data

All manuscripts must include a data availability statement. This statement should provide the following information, where applicable:

- Accession codes, unique identifiers, or web links for publicly available datasets
- A description of any restrictions on data availability
- For clinical datasets or third party data, please ensure that the statement adheres to our policy

Crystal structures are deposited to the PDB: K48-K63-branched Ub3 (PDB ID 7NPO), K48-K63-branched Ub3 in complex with NbSL3 (PDB ID 7NBB), or in complex with NbSL3.3Q (PDB ID 8A67). Peptide searches for mass spectrometry data analysis were conducted using Uniprot Swiss-prot (version release 05/10/2021). Mass-spectrometry data generated in this study are deposited to the PRIDE database (PXD046025). Raw microscopy images have been deposited to Zenodo (10.5281/zenodo.11204921).

Research involving human participants, their data, or biological material

Policy information about studies with human participants or human data. See also policy information about sex, gender (identity/presentation), and sexual orientation and race, ethnicity and racism.

Reporting on sex and gender	This study did not involve human participants. All cell lines used are reported as female in origin.
Reporting on race, ethnicity, or other socially relevant groupings	This study did not involve human participants.
Population characteristics	This study did not involve human participants.
Recruitment	This study did not involve human participants.
Ethics oversight	This study did not involve human participants.

Note that full information on the approval of the study protocol must also be provided in the manuscript.

Field-specific reporting

Please select the one below that is the best fit for your research. If you are not sure, read the appropriate sections before making your selection.

☒ Life sciences ☐ Behavioural & social sciences ☐ Ecological, evolutionary & environmental sciences

For a reference copy of the document with all sections, see nature.com/documents/nr-reporting-summary-flat.pdf

Life sciences study design

All studies must disclose on these points even when the disclosure is negative.

Sample size	Our study did not perform a formal sample size calculation. For mass spectrometry experiments, four replicate samples were used per condition. The sample sizes were determined based on the availability of samples and the feasibility of data collection. We aimed to include as many samples as possible to increase the robustness of our findings.
Data exclusions	No data were excluded from analysis.
Replication	A minimum of two biological replicates were used for all experiments and all were found to be consistent.
Randomization	No grouping was involved in these experiments - no randomisation was conducted as a result.
Blinding	There were no groups, or human or animal participants involved in these experiment - no blinding was required.

Reporting for specific materials, systems and methods

We require information from authors about some types of materials, experimental systems and methods used in many studies. Here, indicate whether each material, system or method listed is relevant to your study. If you are not sure if a list item applies to your research, read the appropriate section before selecting a response.

Materials & experimental systems

n/a	Involved in the study
	☒ Antibodies
	☒ Eukaryotic cell lines
☒	Palaeontology and archaeology
☒	Animals and other organisms
☒	Clinical data
☒	Dual use research of concern
☒	Plants

Methods

n/a	Involved in the study
☒	ChIP-seq
☒	Flow cytometry
☒	MRI-based neuroimaging

Antibodies

Antibodies used	Antibodies used in this study listed below with manufacturer, catalogue numbers, and dilutions used at:<br>Anti-GFP - abcam ab290, 1:2000<br>Anti-GFP - ProteinTech 50430-2-AP, 1:5000<br>Anti-VCP/p97 - ProteinTech 10736-1-AP, 1:4000<br>Anti-ATXN3 - ProteinTech 13505-1-AP, 1:2000<br>Anti-Ubiquitin (P4D1) - Biolegend 646302, 1:2000<br>Anti-Ubiquitin K48-specific (Apu2) - Sigma Aldrich ZRB2150, 1:2000<br>Anti-UBE2N - Invitrogen 37-1100, 1:2000<br>Anti-GAPDH - ProteinTech 10494-1-AP, 1:5000<br>Anti-α-tubulin (DM1A) - CST 3837, 1:5000<br>Anti-Rabbit IgG HRP-conjugated - CST 7074, 1:5000<br>Anti-Mouse IgG HRP-conjugated - CST 7076, 1:5000<br>Anti-Rabbit IgG IRDye800CW-conjugated - LiCor Biosciences 926-32211, 1:15000<br>Anti-Mouse IgG IRDye800CW-conjugated - LiCor Biosciences 926-32210, 1:15000<br>Anti-Rabbit IgG IRDye680RD-conjugated - LiCor Biosciences 926-68073, 1:15000<br>Anti-Mouse IgG IRDye680RD-conjugated - LiCor Biosciences 926-68070, 1:15000
Validation	Anti-ATXN3, anti-UBE2N and anti-p97 antibodies were validated using siRNA knockdown of their respective targets followed by western blotting (anti-ATXN3 - fig 7B, anti-p97 - fig 7B, anti-UBE2N - figE7e)<br>The anti-GFP antibodies were validated using a parental cell line not expressing GFP, and/or a related cell line expressing native-size GFP. In our hands, when used for Western blotting, the abcam antibody gives a non-specific band at the approximate size of native GFP (~27kDa) across both wild-type cell lines used. (anti-GFP ProteinTech - data not shown, anti-GFP abcam -fig E7e)<br>Anti-ubiquitin antibody (P4D1) was validated by comparing untreated and MG132-treated cells in order to detect an increase in ubiquitin levels as suggested by the manufacturer (https://www.biolegend.com/en-gb/products/purified-anti-ubiquitin-antibody-6021 and fig 7a).<br>Anti-ubiquitin K48-specific antibody (Apu2) was validated by western blotting against purified ubiquitin chains incubated ± linkage specific deubiquitinating enzymes (fig 7e).<br>Anti-GAPDH antibody was validated based on size information and supporting western blot images provided on manufacturer's datasheet (https://www.ptglab.com/products/GAPDH-Antibody-81640-5-RR.htm).<br>Anti-α-tubulin antibody was validated based on size information and supporting western blot images provided on manufacturer's datasheet (https://www.cellsignal.com/products/primary-antibodies/a-tubulin-dm1a-mouse-mab/3873)

Eukaryotic cell lines

Policy information about cell lines and Sex and Gender in Research

Cell line source(s)	U2OS - human osteosarcoma, female - Source: ATCC<br>U2OS Flp In Trex - human osteosarcoma, female - Source: MRC PPU<br>U2OS Flp In Trex FRT/TO NbSL18-GFP - human osteosarcoma, female - Source: This study<br>U2OS Flp In Trex FRT/TO NbSL3.3Q-GFP - human osteosarcoma, female - Source: This study<br>HEK293 Flp In Trex - human embryonic kidney, female - Source: ThermoFisher<br>HEK293 Flp In Trex FRT/TO Ub(G76V)-GFP - human embryonic kidney, female - Source: MRC PPU
Authentication	Cell line morphology was visually compared with available images on ATCC website to confirm identity. GFP-tagged cell lines used in this study were validated via a GFP western blot and visually using a Bio-Rad ZOE fluorescent cell imager. No formal authentication was conducted on the parental cell lines used.
Mycoplasma contamination	All cell lines used in this study were routinely tested for mycoplasma by the MRC PPU Unit tissue culture team. No positive tests were recorded for any of the cell lines used in this study.
Commonly misidentified lines<br>(See ICLAC register)	No cell lines from the ICLAC register have been used in this study
