# Supplementary figures and images for: VCP/p97-associated proteins are binders and debranching enzymes of K48–K63-branched ubiquitin chains
Source: Nat Struct Mol Biol. 2024 Jul 8;31(12):1872–87. doi: 10.1038/s41594-024-01354-y (PMC11638074; doi:10.1038/s41594-024-01354-y)

**c** OTULIN requires K33  
to cleave capped M1-Ub2

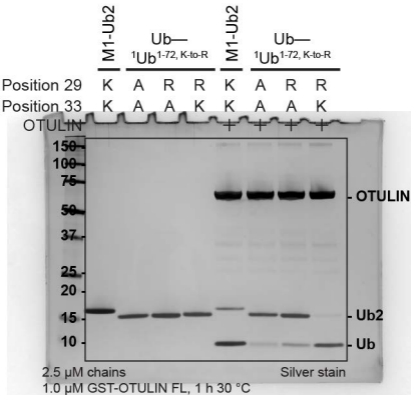

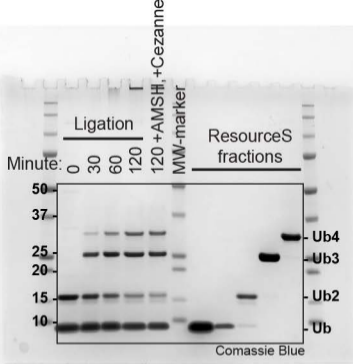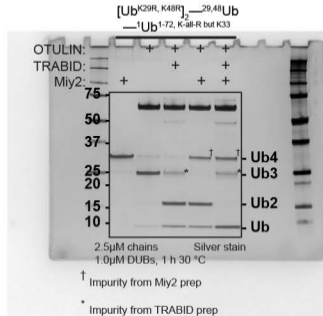

Supplement: Supplementary file 4 — Uncropped gels and blots. [file 41594_2024_1354_MOESM4_ESM.pdf]

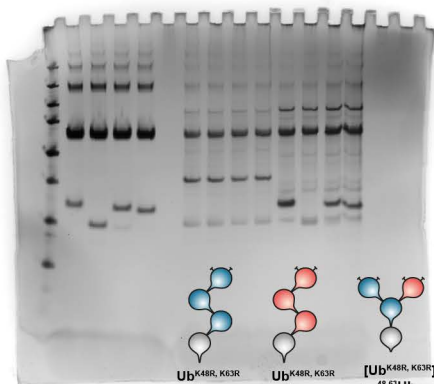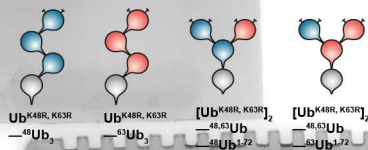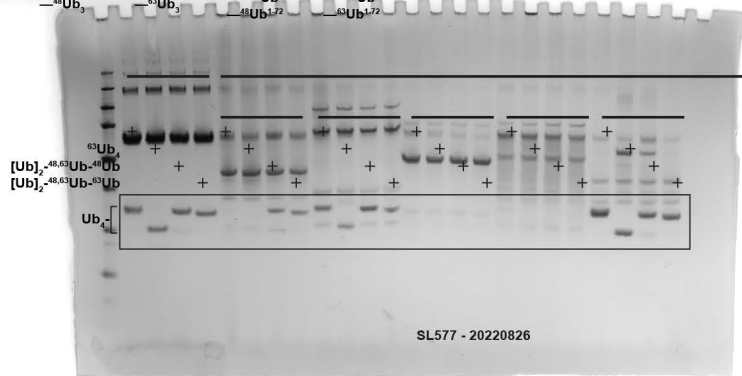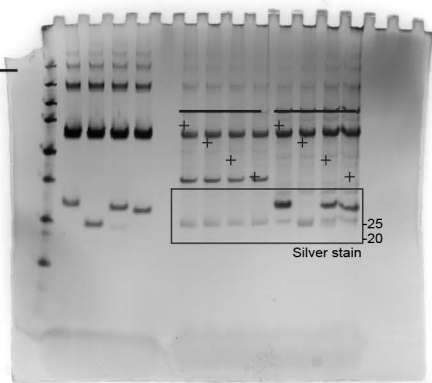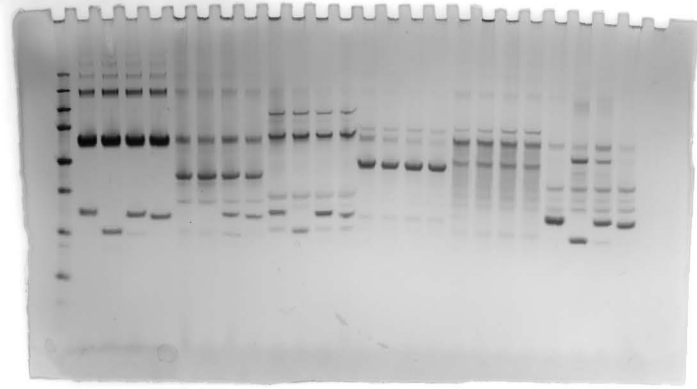

# HALO-RFC1 [190-246]

10% input

pulldown

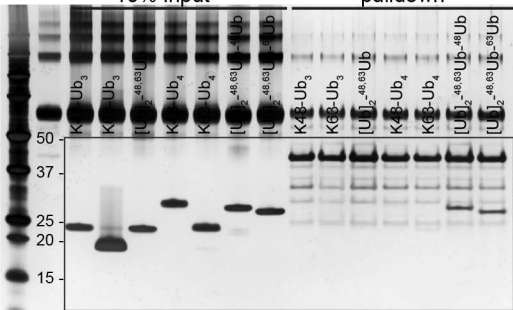

HALO-RFC1

Ub<sub>4</sub>  
Ub<sub>3</sub>

Silver stain

Supplement: Supplementary file 6 — Uncropped gels and blots. [file 41594_2024_1354_MOESM6_ESM.pdf]

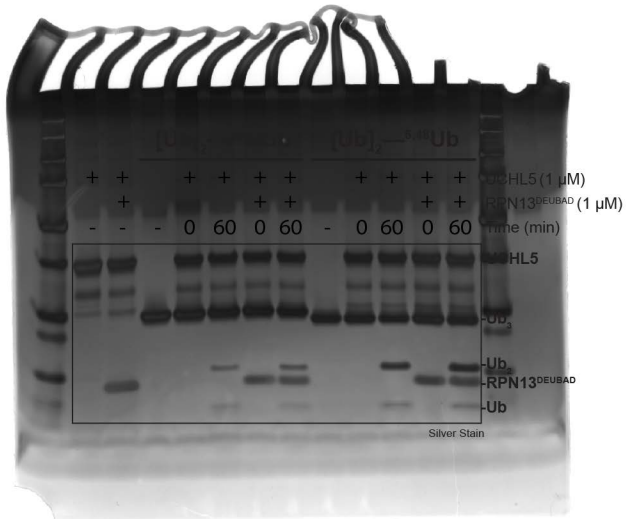

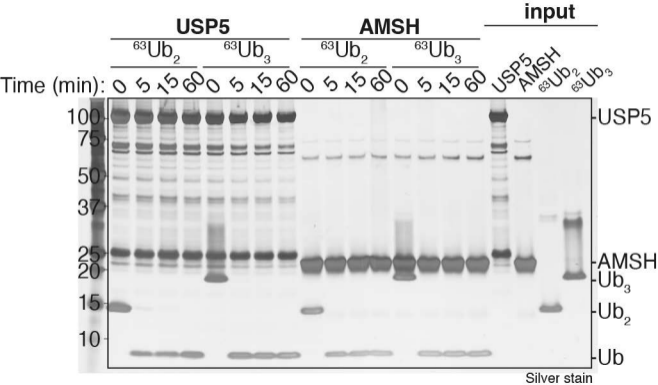

Supplement: Supplementary file 7 — Uncropped gels and blots. [file 41594_2024_1354_MOESM7_ESM.pdf]

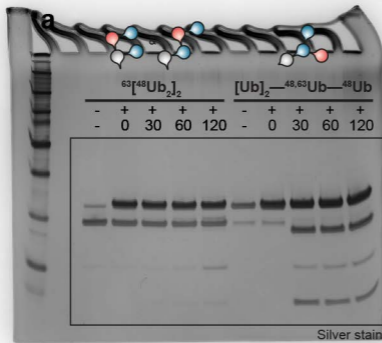

**b**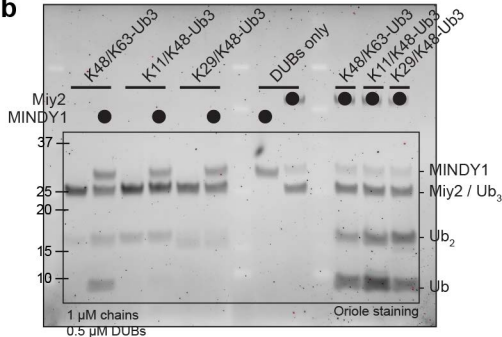

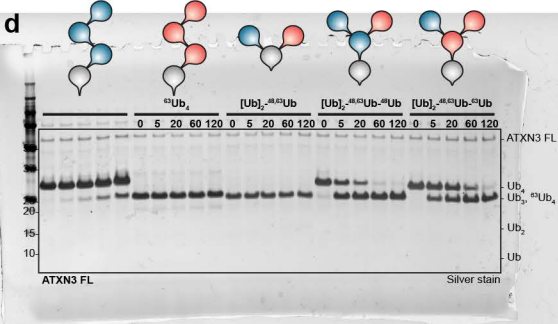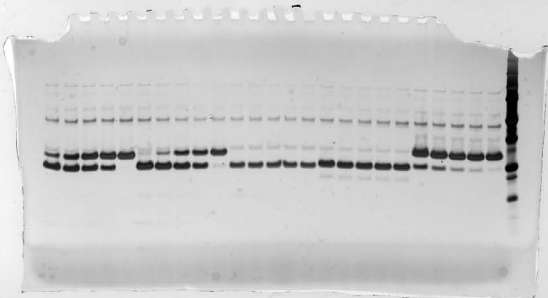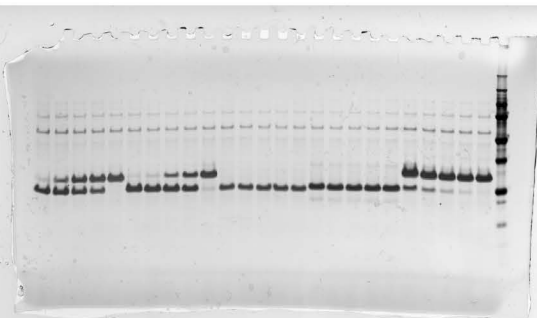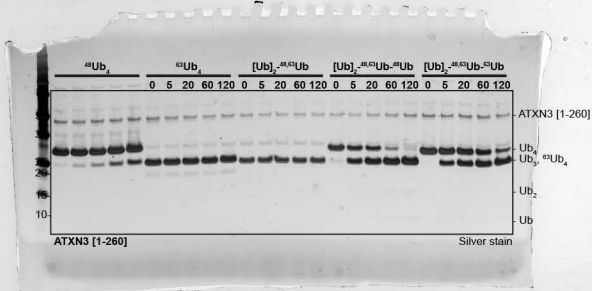

Supplement: Supplementary file 9 — Uncropped gels and blots. [file 41594_2024_1354_MOESM9_ESM.pdf]

**a**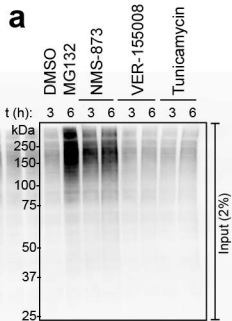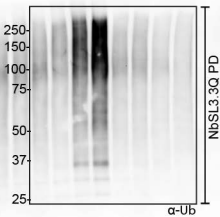

**b**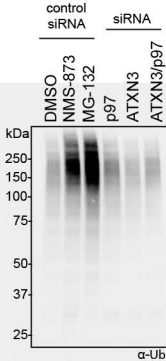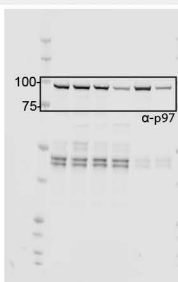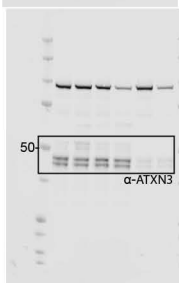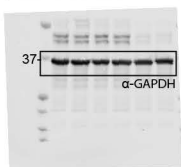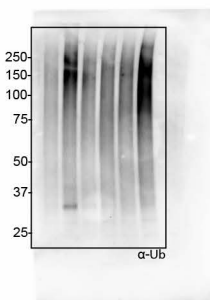

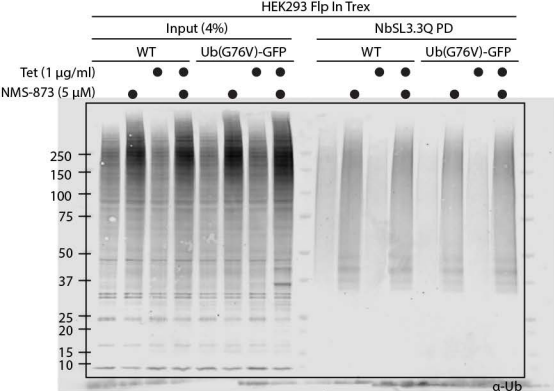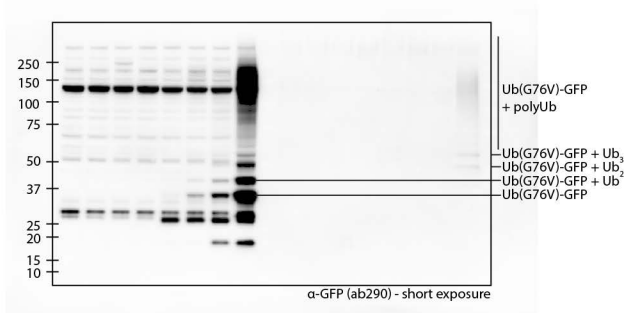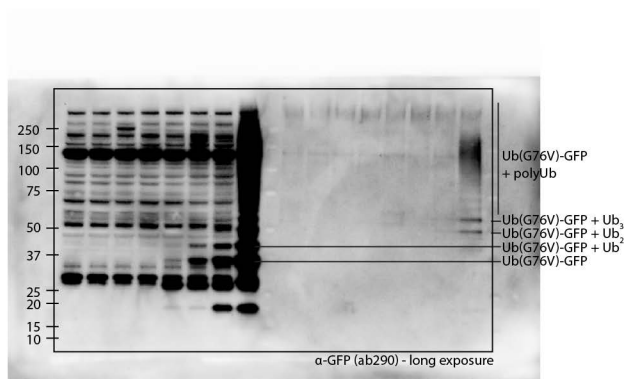

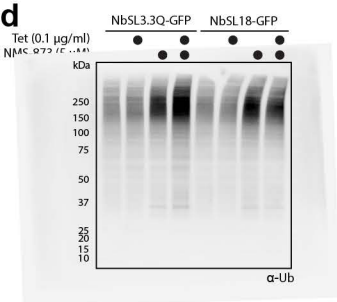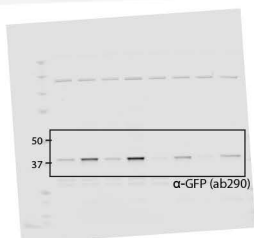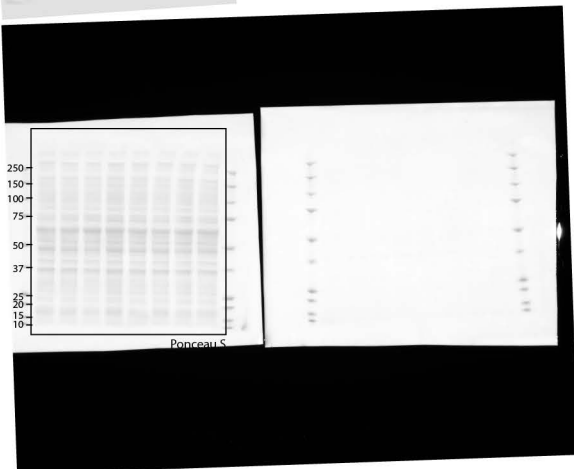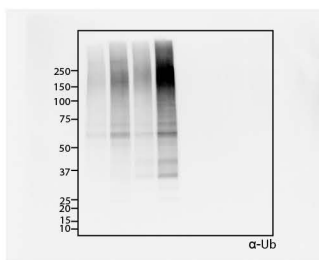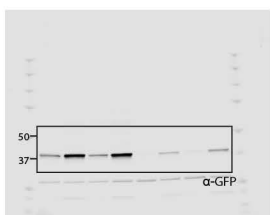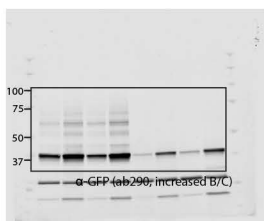

**e**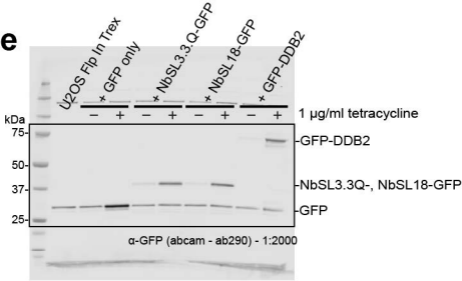

**e**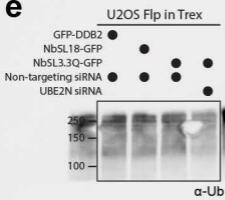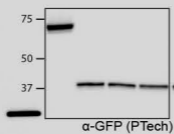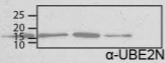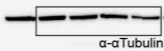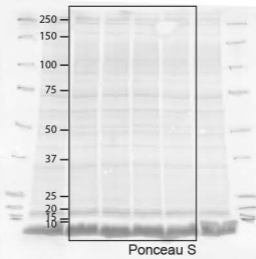

**f**

U2OS Flp in Trex

|                     |   |   |   |   |
|---------------------|---|---|---|---|
| NbSL3.3Q-GFP        | ● | ● | ● | ● |
| NMS-873 (5 $\mu$ M) |   | ● |   | ● |
| Non-targ. siRNA     | ● | ● |   |   |
| ATXN3 siRNA         |   |   | ● | ● |

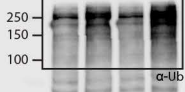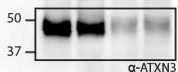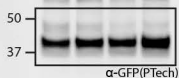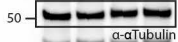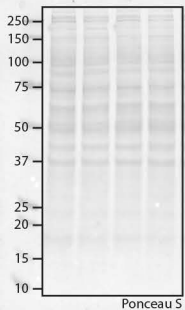

Supplement: Supplementary file 12 — Uncropped gels and blots. [file 41594_2024_1354_MOESM12_ESM.pdf]

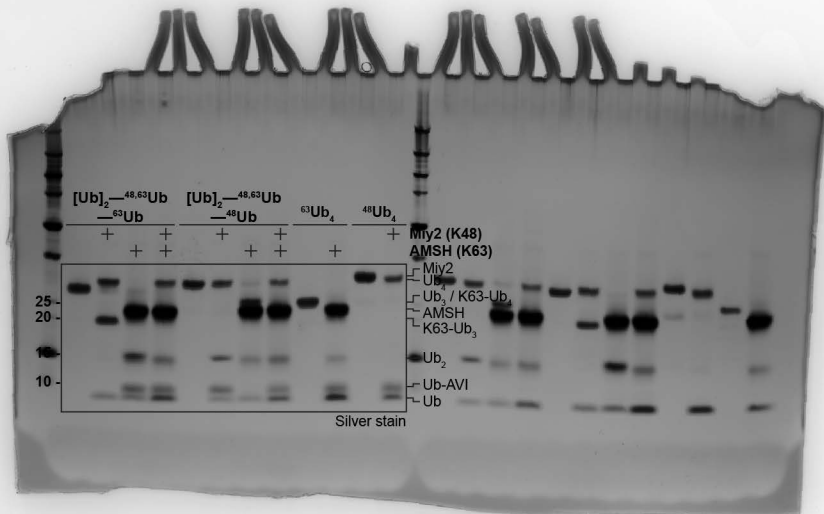

SL567 - 20220918 - gel 2

Supplement: Supplementary file 14 — Uncropped gels and blots. [file 41594_2024_1354_MOESM14_ESM.pdf]

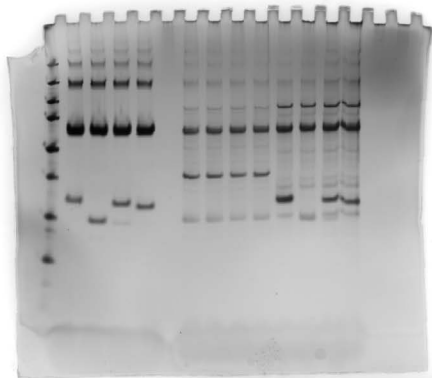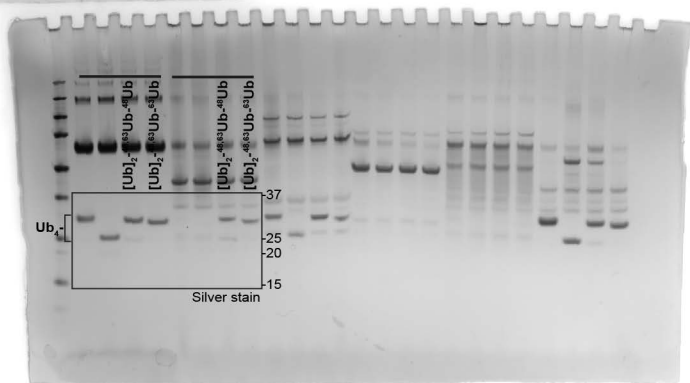

Supplement: Supplementary file 16 — Uncropped gels and blots. [file 41594_2024_1354_MOESM16_ESM.pdf]

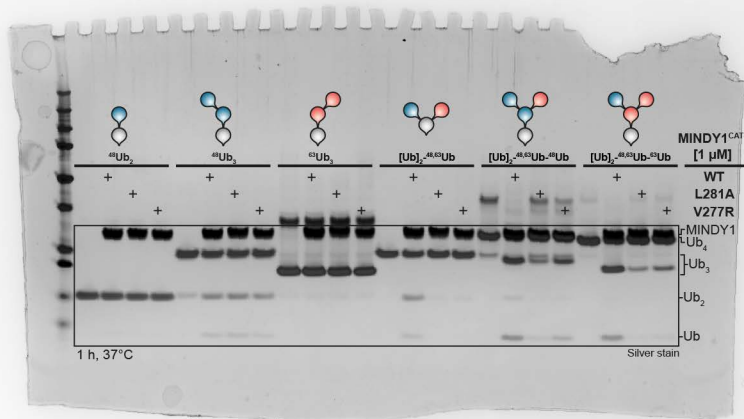

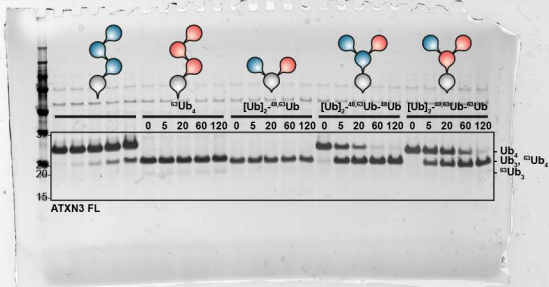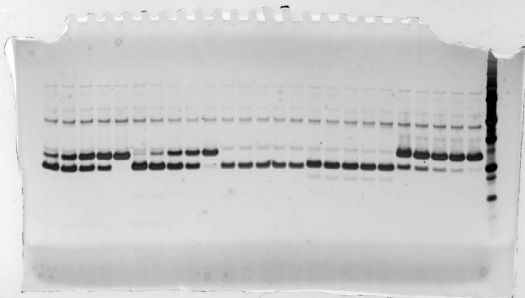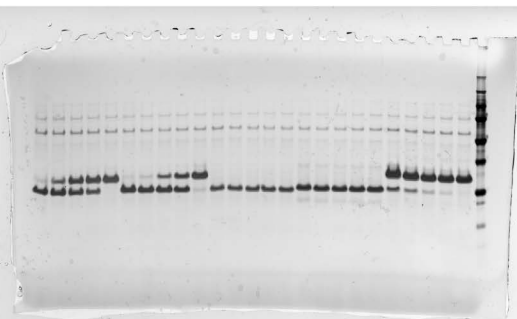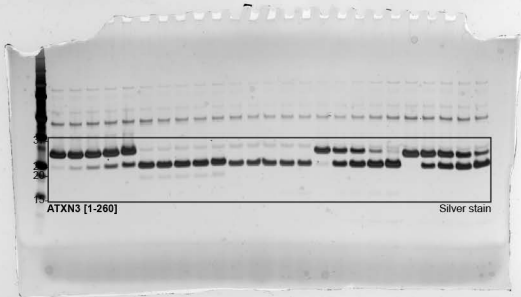

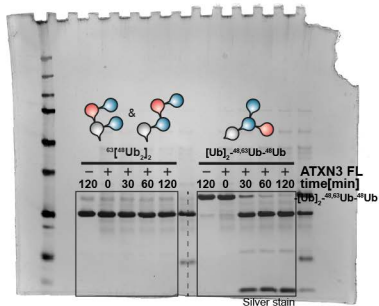

Supplement: Supplementary file 19 — Uncropped gels and blots. [file 41594_2024_1354_MOESM19_ESM.pdf]

d

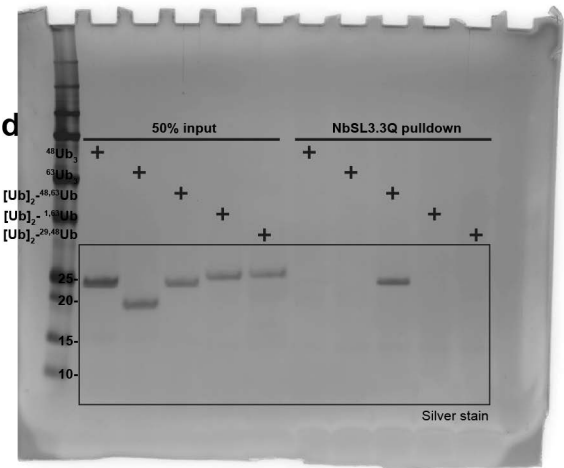

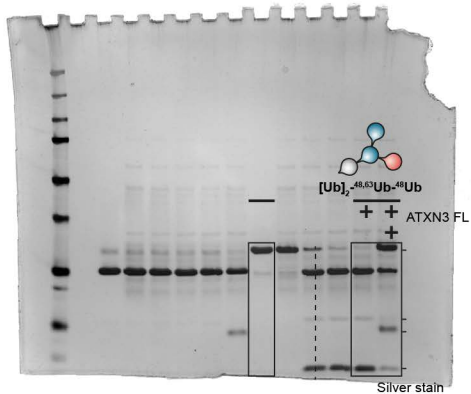

Supplement: Supplementary file 21 — Uncropped gels and blots. [file 41594_2024_1354_MOESM21_ESM.pdf]
