# Supplementary material for: VCP/p97-associated proteins are binders and debranching enzymes of K48–K63-branched ubiquitin chains
Source: Nat Struct Mol Biol. 2024 Jul 8;31(12):1872–87. doi: 10.1038/s41594-024-01354-y (PMC11638074; doi:10.1038/s41594-024-01354-y)
Supplement: Supplementary file 5 — Uncropped gels and blots. [file 41594_2024_1354_MOESM5_ESM.pdf]

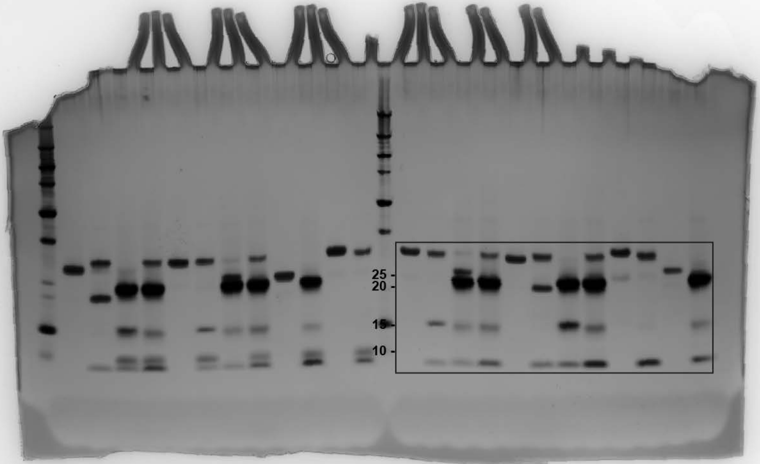

SL567 - 20220918 - gel 2

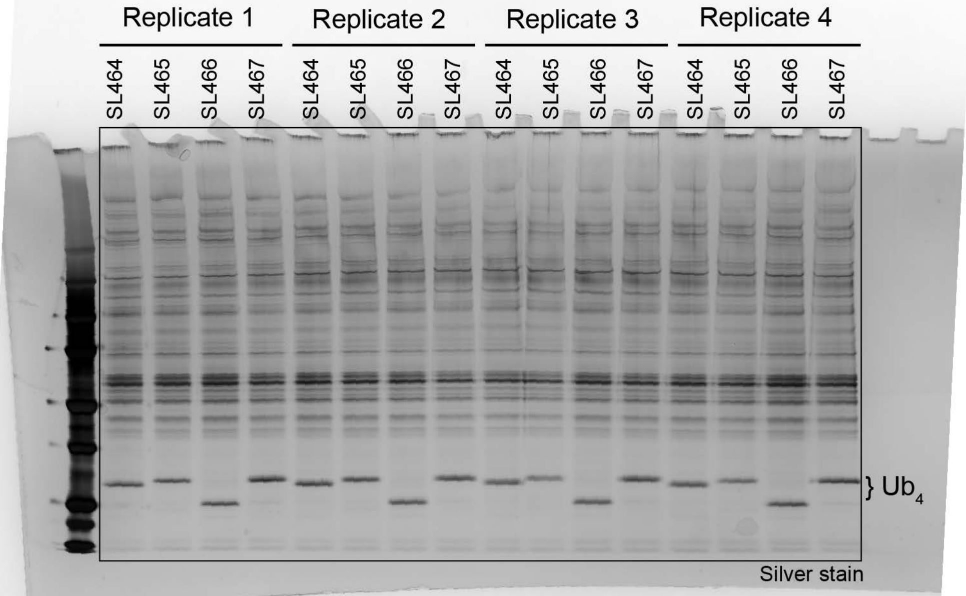

SL484 - 20220217 - Pulldown with branched/unbranched K48/K63 chains from U2OS cell lysate
