# Supplementary material for: VCP/p97-associated proteins are binders and debranching enzymes of K48–K63-branched ubiquitin chains
Source: Nat Struct Mol Biol. 2024 Jul 8;31(12):1872–87. doi: 10.1038/s41594-024-01354-y (PMC11638074; doi:10.1038/s41594-024-01354-y)
Supplement: Supplementary file 10 — Uncropped gels and blots. [file 41594_2024_1354_MOESM10_ESM.pdf]

a

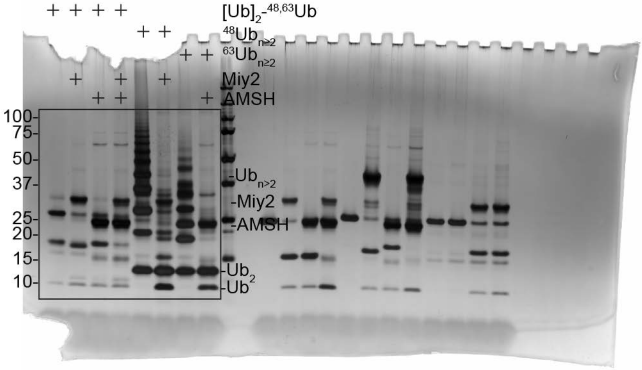

SL567- gel 3 - silver stain

b

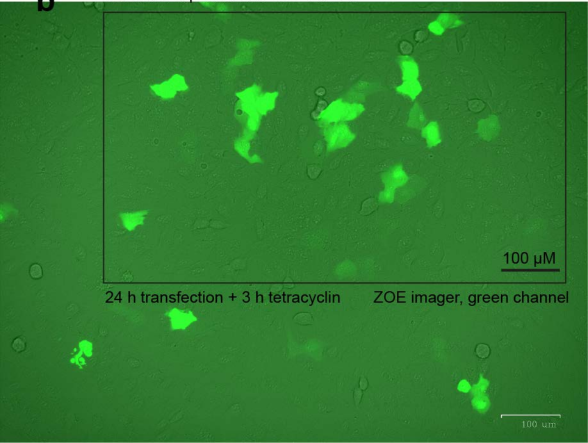
Transient expression of GFP-NbSL3
100 µM
24 h transfection + 3 h tetracyclin
ZOE imager, green channel
100 um
