# Supplementary material for: VCP/p97-associated proteins are binders and debranching enzymes of K48–K63-branched ubiquitin chains
Source: Nat Struct Mol Biol. 2024 Jul 8;31(12):1872–87. doi: 10.1038/s41594-024-01354-y (PMC11638074; doi:10.1038/s41594-024-01354-y)
Supplement: Supplementary file 23 — Uncropped gels and blots. [file 41594_2024_1354_MOESM23_ESM.pdf]

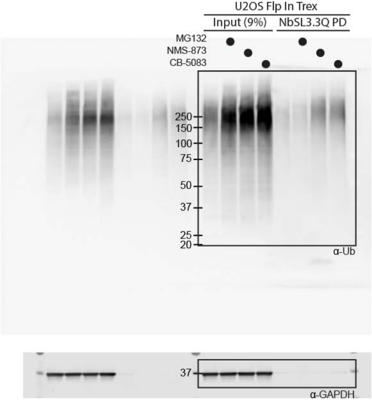

U2OS Flp In Trex
Input (9%)
NbSL3.3Q PD
MG132
NMS-873
CB-5083
250
150
100
75
50
37
25
20
α-Ub
37
α-GAPDH

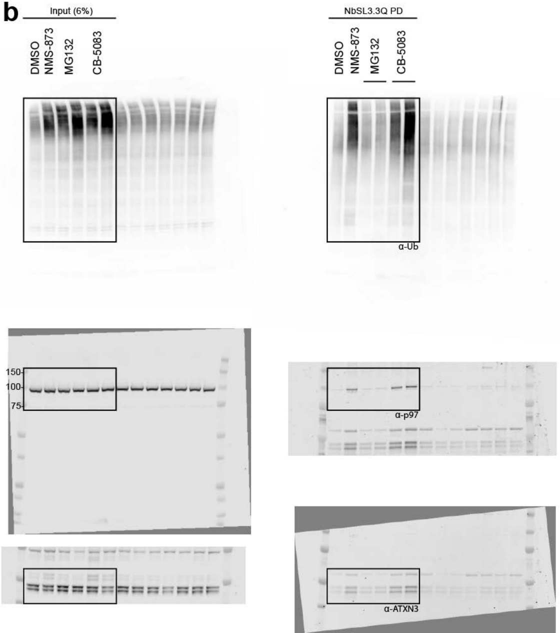
b
Input (6%)
DMSO
NMS-873
MG132
CB-5083
NbSL3.3Q PD
DMSO
NMS-873
MG132
CB-5083
α-Ub
150
100
75
α-p97
α-ATXN3

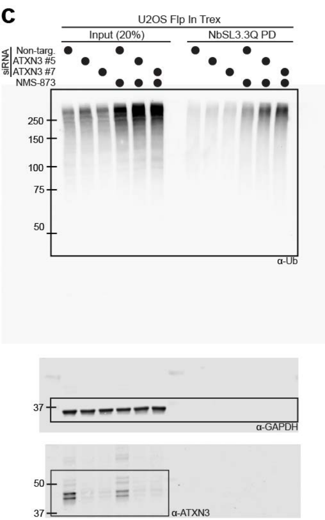
c
U2OS Flp In Trex
Input (20%)
NbSL3.3Q PD
siRNA
Non-targ.
ATXN3 #5
ATXN3 #7
NMS-873
250
150
100
75
50
α-Ub
37
α-GAPDH
50
37
α-ATXN3

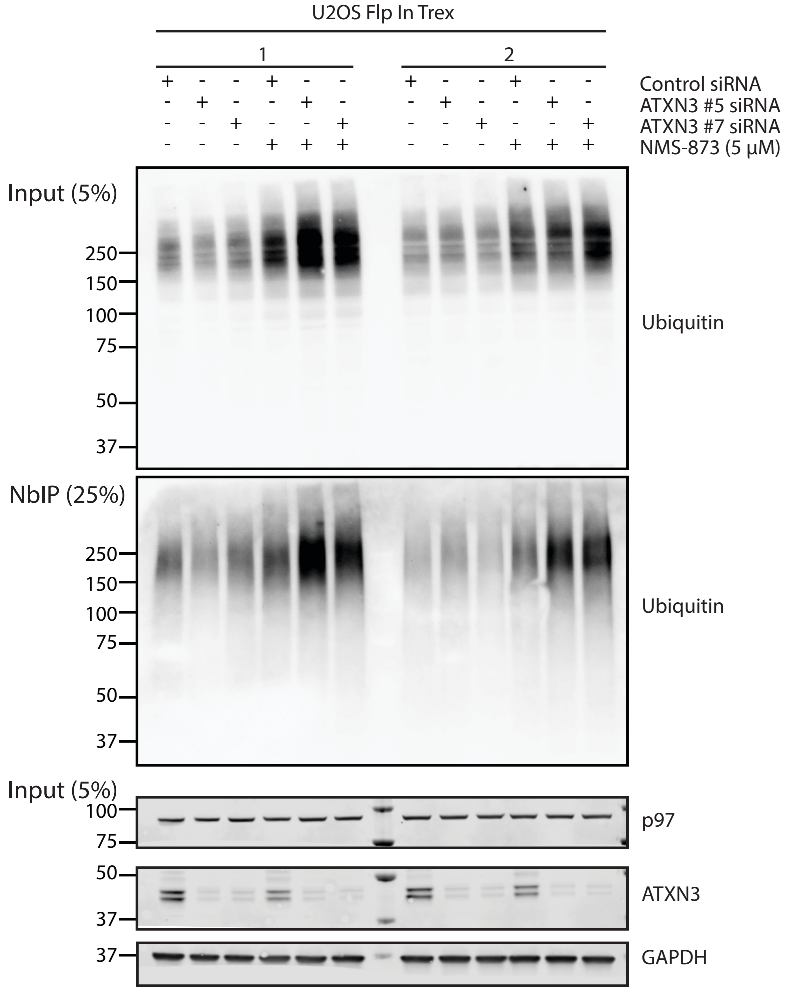

U2OS Flp In Trex
1
2
Control siRNA
ATXN3 #5 siRNA
ATXN3 #7 siRNA
NMS-873 (5 µM)
Input (5%)
NbIP (25%)
Ubiquitin
p97
ATXN3
GAPDH
250
150
100
75
50
37

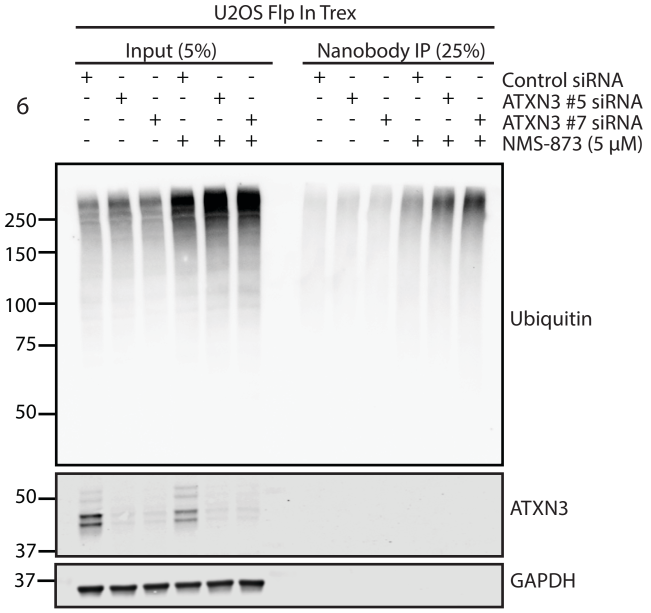

6
U2OS Flp In Trex
Input (5%)
Nanobody IP (25%)
Control siRNA
ATXN3 #5 siRNA
ATXN3 #7 siRNA
NMS-873 (5 µM)
Ubiquitin
ATXN3
GAPDH
250
150
100
75
50
37

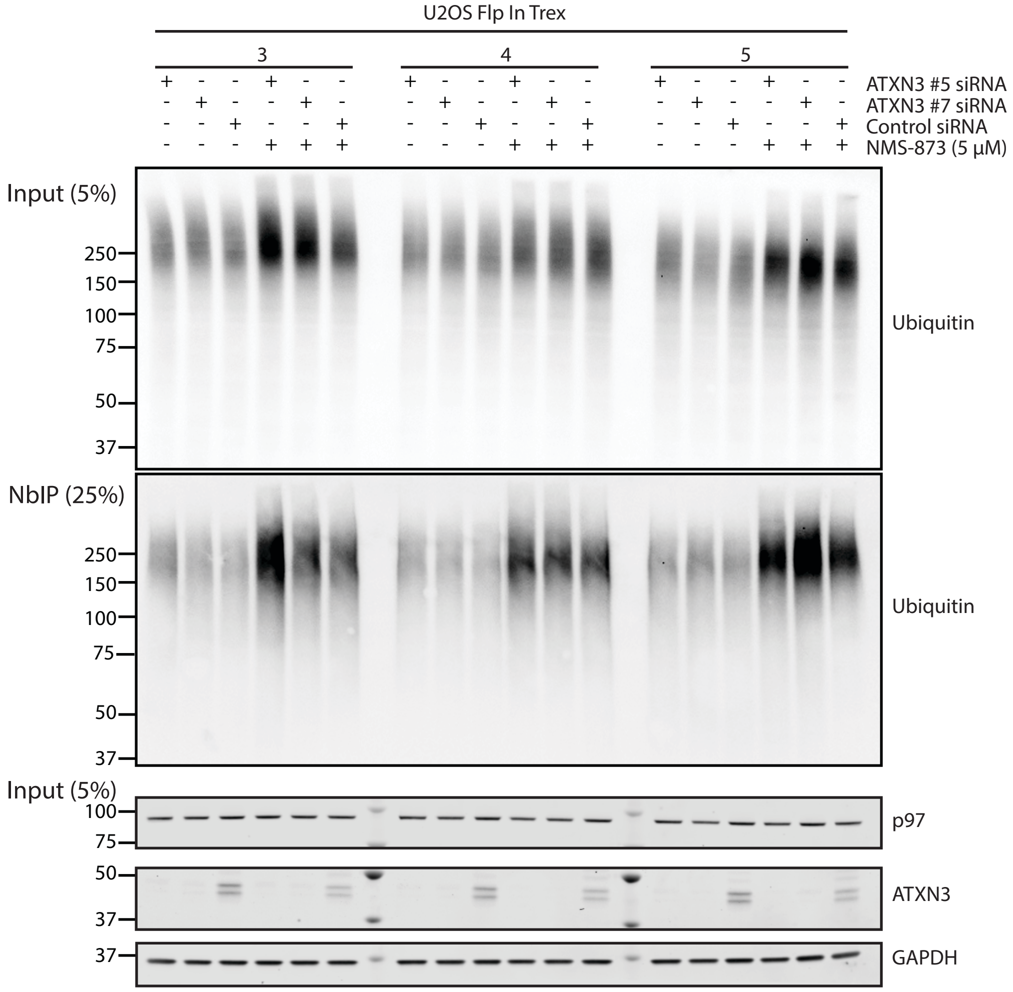

U2OS Flp In Trex
3
4
5
ATXN3 #5 siRNA
ATXN3 #7 siRNA
Control siRNA
NMS-873 (5 µM)
Input (5%)
NbIP (25%)
Ubiquitin
p97
ATXN3
GAPDH
250
150
100
75
50
37

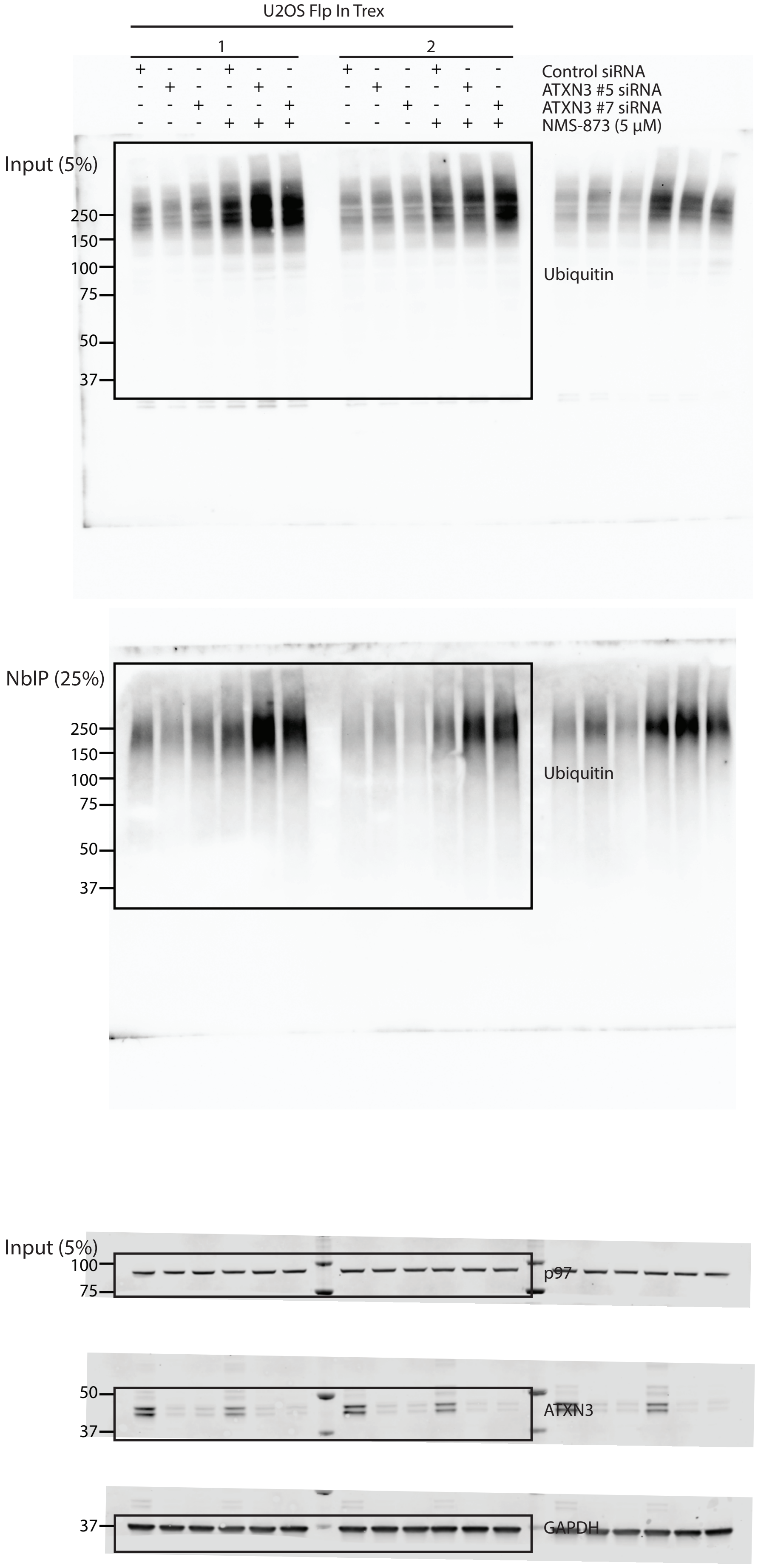
U2OS Flp In Trex
1
2
Control siRNA
ATXN3 #5 siRNA
ATXN3 #7 siRNA
NMS-873 (5 µM)
Input (5%)
250
150
100
75
50
37
Ubiquitin
NbIP (25%)
Ubiquitin
Input (5%)
p97
ATXN3
GAPDH

6

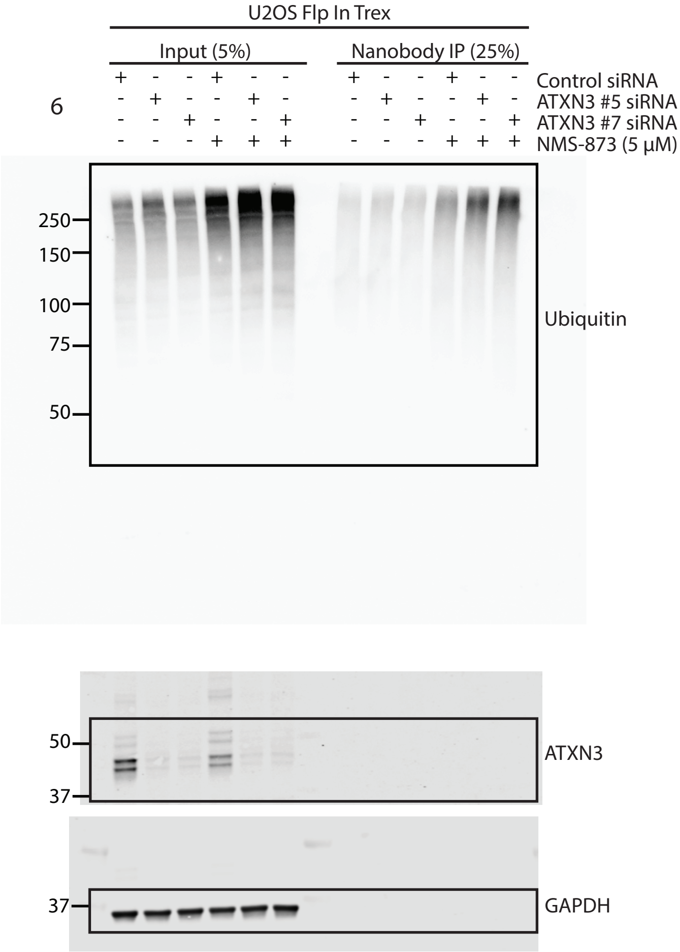
U2OS Flp In Trex
Input (5%)
Nanobody IP (25%)
Control siRNA
ATXN3 #5 siRNA
ATXN3 #7 siRNA
NMS-873 (5 µM)
250
150
100
75
50
Ubiquitin
50
37
ATXN3
37
GAPDH

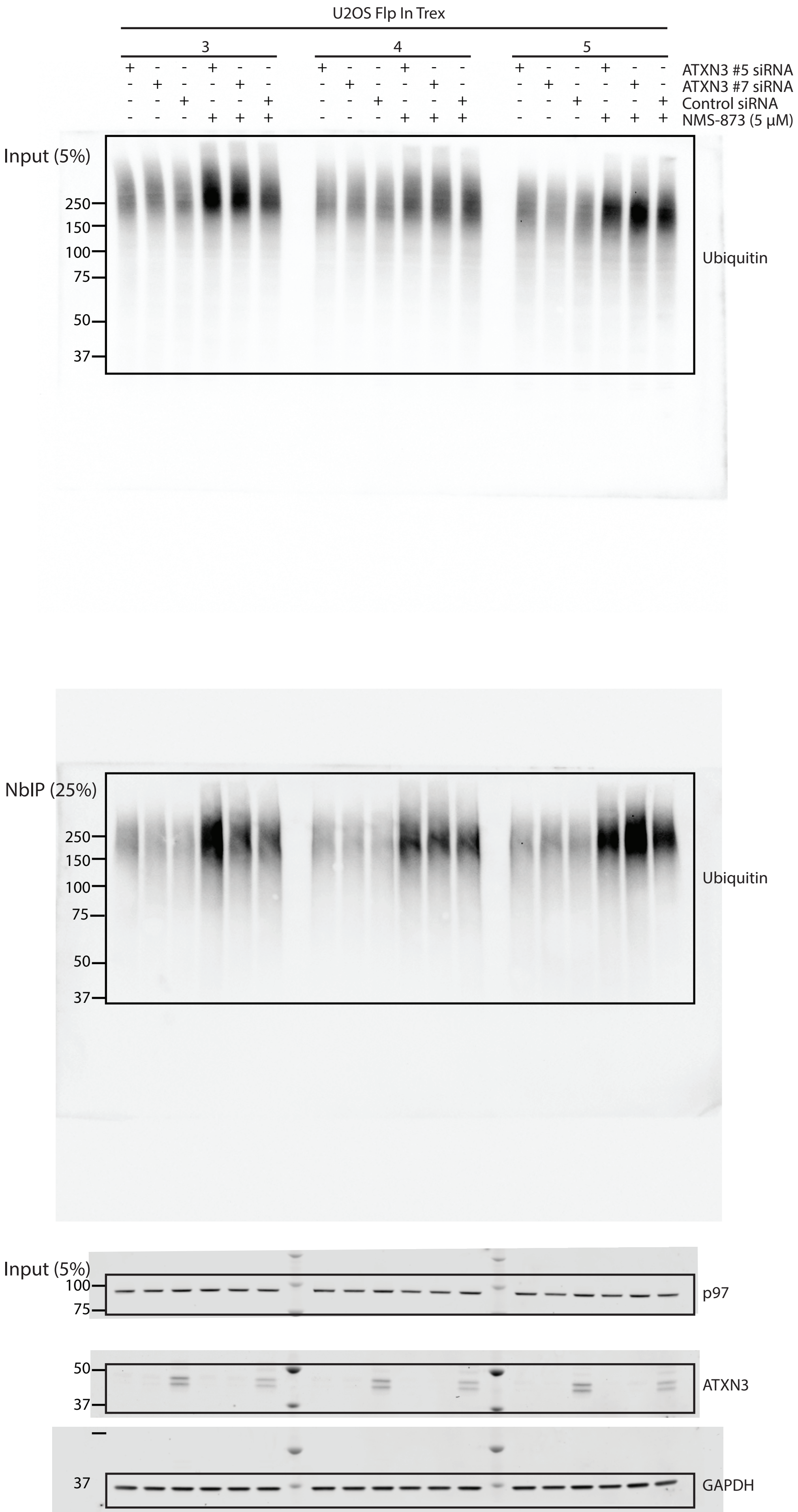
U2OS Flp In Trex
3
4
5
ATXN3 #5 siRNA
ATXN3 #7 siRNA
Control siRNA
NMS-873 (5 µM)
Input (5%)
250
150
100
75
50
37
Ubiquitin
NbIP (25%)
250
150
100
75
50
37
Ubiquitin
Input (5%)
100
75
p97
50
37
ATXN3
37
GAPDH

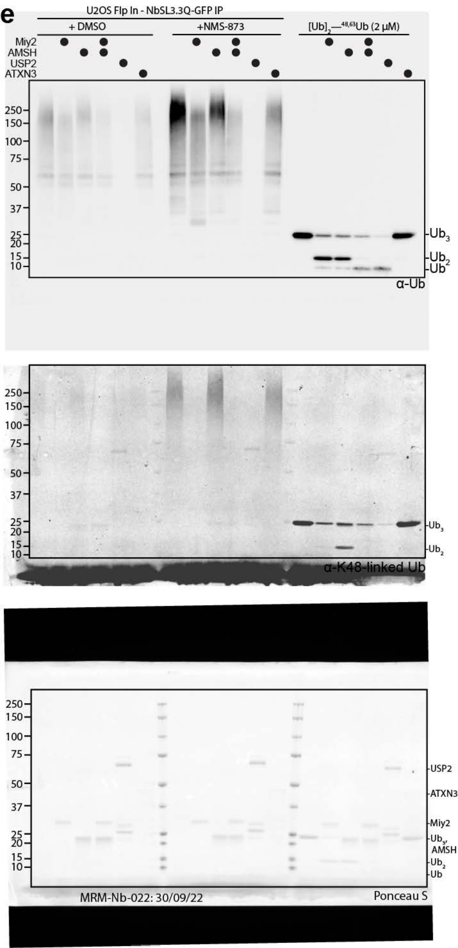
e
U2OS Flp In - NbSL3.3Q-GFP IP
+ DMSO
+NMS-873
[Ub]2—48,63Ub (2 μM)
Miy2
AMSH
USP2
ATXN3
250
150
100
75
50
37
25
20
15
10
-Ub3
-Ub2
-Ub
α-Ub
250
150
100
75
50
37
25
20
15
10
-Ub3
-Ub2
α-K48-linked Ub
250
150
100
75
50
37
25
20
15
10
-USP2
-ATXN3
-Miy2
-Ub3, AMSH
-Ub2
-Ub
MRM-Nb-022: 30/09/22
Ponceau S
